# EFAR-MMLA: An Evaluation Framework to Assess and Report Generalizability of Machine Learning Models in MMLA

**DOI:** 10.3390/s21082863

**Published:** 2021-04-19

**Authors:** Pankaj Chejara, Luis P. Prieto, Adolfo Ruiz-Calleja, María Jesús Rodríguez-Triana, Shashi Kant Shankar, Reet Kasepalu

**Affiliations:** 1 School of Digital Technologies, Tallinn University, 10120 Tallinn, Estonia; mjrt@tlu.ee (M.J.R.-T.); shashik@tlu.ee (S.K.S.); 2 School of Educational Sciences, Tallinn University, 10120 Tallinn, Estonia; lprisan@tlu.ee (L.P.P.); reetkase@tlu.ee (R.K.); 3 GSIC-EMIC Group, University of Valladolid, 47011 Valladolid, Spain; adolfo@gsic.uva.es

**Keywords:** Multimodal Learning Analytics, MMLA, face-to-face collaboration, machine learning, generalizability, evaluation framework, reporting

## Abstract

Multimodal Learning Analytics (MMLA) researchers are progressively employing machine learning (ML) techniques to develop predictive models to improve learning and teaching practices. These predictive models are often evaluated for their generalizability using methods from the ML domain, which do not take into account MMLA’s educational nature. Furthermore, there is a lack of systematization in model evaluation in MMLA, which is also reflected in the heterogeneous reporting of the evaluation results. To overcome these issues, this paper proposes an evaluation framework to assess and report the generalizability of ML models in MMLA (EFAR-MMLA). To illustrate the usefulness of EFAR-MMLA, we present a case study with two datasets, each with audio and log data collected from a classroom during a collaborative learning session. In this case study, regression models are developed for collaboration quality and its sub-dimensions, and their generalizability is evaluated and reported. The framework helped us to systematically detect and report that the models achieved better performance when evaluated using hold-out or cross-validation but quickly degraded when evaluated across different student groups and learning contexts. The framework helps to open up a “wicked problem” in MMLA research that remains fuzzy (i.e., the generalizability of ML models), which is critical to both accumulating knowledge in the research community and demonstrating the practical relevance of these techniques.

## 1. Introduction

Multimodal Learning Analytics (MMLA) extends Learning Analytics (LA) by gathering data from digital and physical spaces to gain a holistic picture of the learning process [1,2,3]. Besides traditional digital logs, MMLA researchers have used a variety of sensors (e.g., cameras, microphones, eye-trackers) [4,5,6] to gather data about learning activities, student behavior, physiology and the learning environment [7]. Establishing the connection between these data sources and learning can also help to bridge the gap between learning science and machine learning (ML) techniques [4,7]. For example, Spikol et al. [8] identified the distance between participants’ hands during collaborative learning sessions as a proxy for collaboration behavior using ML. Such uses of ML pave the way for automated systems to support teaching and learning using multimodal data.

ML is defined as “computational methods using experience to improve performance or make accurate predictions” [9]. In MMLA, experience refers to the data related to students, learning or the learning environment. MMLA researchers have used ML to build predictive models for a variety of learning constructs; e.g., attention, the social plane of interaction, math expertise, etc. [4]. These predictive models go through a development and evaluation process [4,9] to test their readiness before the final deployment in the real-world. This process involves the following main steps:

- The development step (also known as model training) fits the ML models to the available data and optionally finds hyper-parameters. Hyper-parameters are the tuning parameters of the ML models [10] (e.g., the number of hidden layers in a neural network) that need to be configured before model training [11].
- After the model training, the evaluation step assesses the models’ performance. The model evaluation serves different purposes depending on the goal of the ML model in MMLA. For instance, we use model evaluation to find the best-fitted model to the data when we develop the model to identify learning indicators (e.g., data features with high predictive power). Meanwhile, in the case of building a predictive ML model, the evaluation step assesses the performance on unseen data [9]. The model’s ability to perform on unseen data is also referred to as generalizability [10,12].

MMLA researchers have assessed and reported the performance of their ML models in different ways [13,14]. Moreover, most authors have used conventional evaluation methods that do not provide a measure of generalizability relevant to the MMLA field. For example, the cross-validation evaluation method helps us to understand the model’s performance on random folds of data. However, it does not offer information about the model’s performance on data arising from different groups of students or learning contexts. The lack of common practices while evaluating and reporting ML performance complicates the comparison of current MMLA ML proposals. Moreover, the insufficiency of conventional methods in assessing the generalizability on MMLA relevant levels hinders the community from understanding the current state of MMLA in achieving solutions that are of practical relevance.

This paper proposes an evaluation framework to assess and report on the generalizability of ML models in MMLA (EFAR-MMLA). This paper also presents a case study to illustrate how to apply the framework and how it can help to reflect on the generalizability of ML models. This case study reports the evaluation of ML models to estimate collaboration in a face-to-face classroom setting, using audio and log data. This case study also investigates the performance of ML models in estimating dimensions of collaboration.

The rest of the paper is structured in five sections. The next section offers an overview of model evaluation methods in ML and MMLA. Section 3 presents EFAR-MMLA. Section 4 presents a case study using EFAR-MMLA. Section 5 discusses the applications and limitations of the EFAR-MMLA. Finally, Section 6 concludes the paper with future research plans.

## 2. Model Evaluation in Machine Learning and Multimodal Learning Analytics

To make this section self-contained, Section 2.1 briefly summarizes the model evaluation methods from the ML domain before reviewing their use by MMLA researchers in Section 2.2.

### 2.1. Model Evaluation in ML

**Figure 1:**
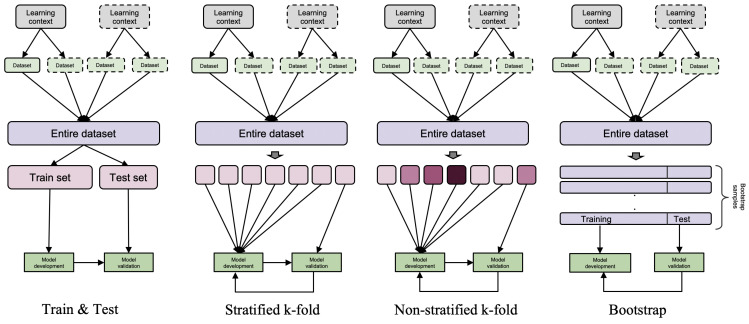
ML model evaluation methods.

There are multiple methods available in the ML domain to perform model evaluation; e.g., hold-out, cross-validation and bootstrap [10]. These methods differ in the way the dataset is partitioned for training and testing purposes. For example, in hold-out, data are randomly split into two parts; namely, the training and test set [10] (shown in Figure 1). The training set is used for the model development, while the test set is used to assess the model’s performance. On the other hand, cross-validation methods (e.g., k-fold with and without stratification) [15] split the dataset into multiple partitions and involve multiple repetitions of training and testing. For example, the k-fold cross-validation method randomly divides the dataset into k equal-sized partitions (Figure 1). One of those partitions is kept for the model evaluation, and the rest of the partitions are used for the model development. This process is repeated k times, and each time a different partition is used for evaluation. The stratified version of k-fold partitions the dataset in such a way that it preserves the representativeness of the original dataset. In other words, each partition after splitting approximately contains the same data distribution or same proportion of labels as in the original dataset. The leave-one out evaluation is a special case of k-fold cross-validation where k equals the number of instances in the dataset. The splitting in hold-out and cross-validation methods is done exclusively; e.g., each data instance belongs to a single set (if hold-out) or partition (if cross-validation). On the contrary, the bootstrap method generates new data from the available dataset using repeated sampling with replacement [10,16]. The models are then developed and assessed on those bootstrapped samples.

### 2.2. Model Evaluation in MMLA

MMLA researchers have developed ML models for various learning constructs (see Table 1). The majority of MMLA researchers have used a similar process to develop their ML model: the specification of the type of a problem (e.g., estimating learning construct on future data or identifying patterns in the data collected), collection of data, data annotation if the problem is the development of a predictive model to estimate a leaning construct, feature extraction, feature selection, model training and then model evaluation [13,14,17].

Table 1 shows that a variety of evaluation methods are used in MMLA. For example, Giannakos et al. [18] used hold-out to evaluate their models’ performance in estimating skill acquisition; in contrast, the majority of researchers have used k-fold cross-validation with varying values of k (e.g., 4, 5 and 10) [17,19,20]. Most MMLA researchers using ML have used data from a single dataset (or data distribution) for model evaluation [13,21,22], and this dataset is often collected from a single physical space with a particular set of people or learning activities. However, research in ML suggests testing data from various distributions (or different studies [23]) to obtain a reliable estimate of generalizability [12]. A few MMLA researchers [5,17,24] have taken this into consideration while assessing their models. They have gone a step further by evaluating their model’s generalizability across student groups [24], datasets [17] and contexts [5]. For instance, Sharma et al. [5] investigated their models’ performance across different tasks and contexts using leave-one study out evaluation. Prieto et al. [17] used leave-one session out evaluation to test the generalizability of their model across datasets.

A difference is also found in the way MMLA researchers have reported their model’s performances. The majority of researchers only reported their models’ mean performance while using cross-validation techniques [21,22]. However, mean performance alone does not offer information on the reliability of a reported model’s performance. Therefore, some researchers [8,18,19] have also reported variations in their model’s performance (e.g., using the variance [8,19] and 95% confidence interval [18]). A very small number of researchers have reported their hyper-parameter searching strategy [25]. MMLA researchers [22,24] have also used a baseline performance to show the added value of the built model over a chance model or a model with a fixed strategy; e.g., a majority classifier.

From the analysis of the ML models’ evaluation and performance reported in the aforementioned MMLA works, we have identified three main problems.

- The widely used cross-validation methods are limited in terms of providing a generalizability assessment [23]. The use of these methods is neither recommended for model comparison nor selection purposes [30,31,32]. The performance measure obtained from a cross-validation step, which is also used for hyper-parameter tuning, is found to be significantly biased [30].
- The model evaluation methods from ML do not assess generalizability at the levels relevant to the MMLA field. For example, the cross-validation method assesses the model’s generalizability across folds containing random data from the dataset. This kind of evaluation cannot offer information on how the model will perform on data from different students or classrooms.
- The heterogeneous approach to performance reporting hinders the community from accumulating knowledge regarding the maturity of ML in the field. MMLA researchers are employing different baselines and often reporting the model’s average performance without giving an uncertainty measure [13,28,29]. The use of baseline performance only offers a lower bound of performance, which is not sufficient to understand the practical value of the model.

The availability of different evaluation methods makes it difficult for non-ML researchers to select the most appropriate one. Furthermore, there is a need to systematize the ML model evaluation and performance reporting in MMLA. In other fields, various solutions have been proposed to address the lack of standard model evaluation methods [23,33]. For instance, in clinical research, Justice et al. [33] proposed a framework for ML model evaluation at various generalizability levels. These generalizability levels are based on several factors; e.g., time, geography, patient’s disease level, etc. The differences between these research fields and MMLA complicates the adoption of such a framework to MMLA. For example, the ML evaluation framework in clinical research considers a dimension that may bring changes in the data distribution of future data to identify different levels of generalizability. As an illustrative case, the developed clinical ML models can be applied for estimation purposes to patients who are from different locations than those who were involved in the model’s training. Thus, geographical generalizability is one of the levels in their evaluation framework. Similarly, other levels identified in the clinical research domain to evaluate models are mainly dependent on patients only. In contrast, in the education domain, learners are not considered in isolation; rather, they are often considered within a certain learning context (e.g., classroom, school, etc.). Therefore, additional dimensions for the learning context are relevant when considering the MMLA domain. Such considerations have also been recommended by Cronbach et al., who stated the need to consider students as being nested within classrooms and schools while assessing the generalizability of students’ achievement [34]. If we were to follow the same approach as clinical research, we would get a multitude of generalizability levels (e.g., across learning content, across learning spaces, across teaching practices, across different students) in MMLA, giving an overly complex framework with which to work. Instead, we should utilize the existing evaluation practices that are already available in the domain to systematize the evaluation process. For example, MMLA researchers have used evaluation methods to assess generalizability at various levels; e.g., across groups. However, these practices are not standardized in the domain. Thus, we foresee a need to bring systematization into the MMLA field for ML model evaluation. While existing frameworks could partially inform MMLA practices, MMLA researchers need a domain-specific framework to assess the generalizability of ML models. The following section proposes a framework to address this need.

## 3. EFAR-MMLA

**Figure 2:**
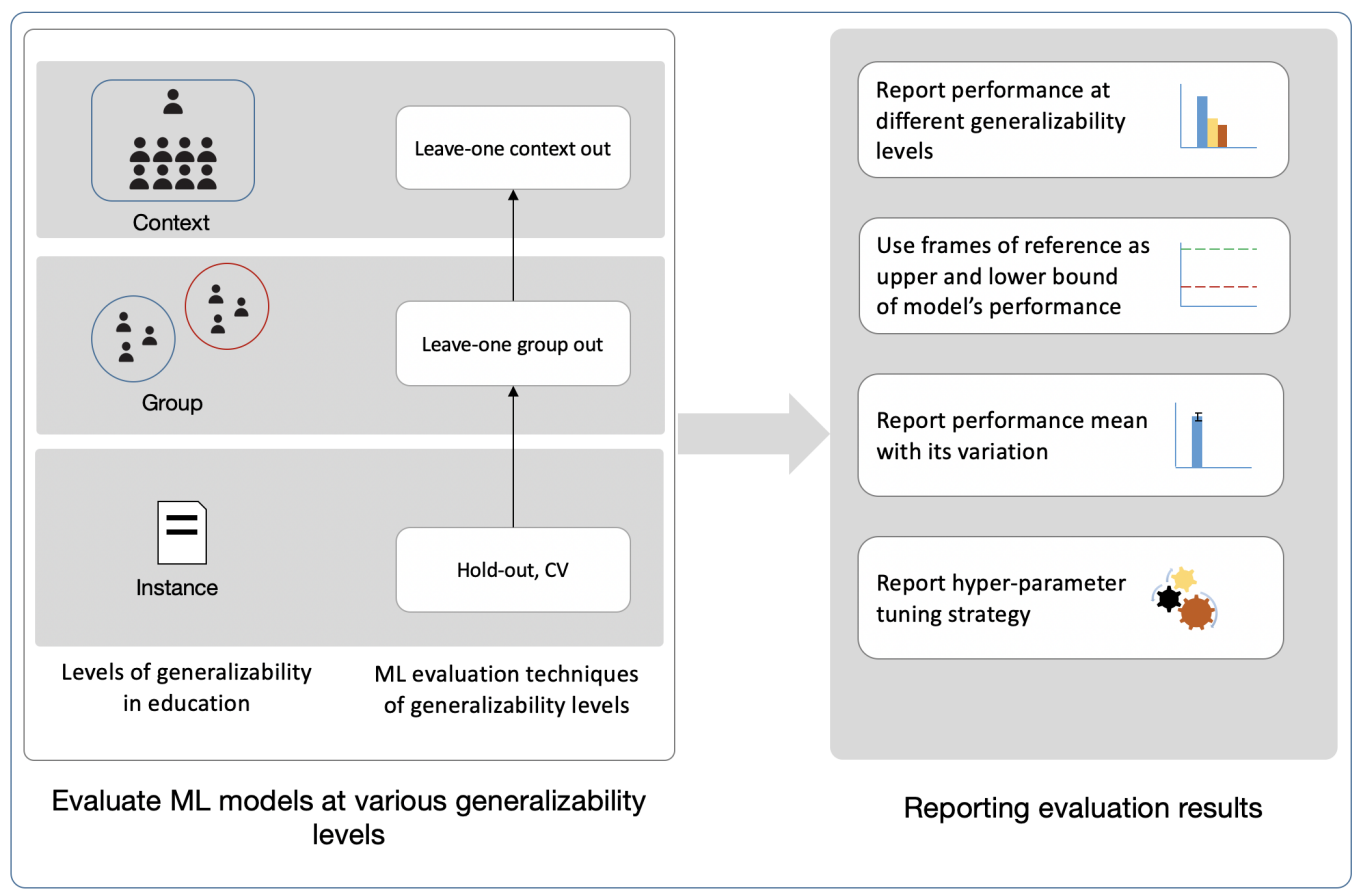
EFAR-MMLA: Evaluation Framework for Assessing and Reporting Generalizability of ML models in MMLA.

We propose an evaluation framework for assessing and reporting MMLA ML models (EFAR-MMLA). The framework contains two components: model evaluation (Section 3.1) and performance reporting (Section 3.2) at different generalizability levels (see Figure 2).

### 3.1. Model Evaluation at Different Generalizability Levels

**Figure 3:**
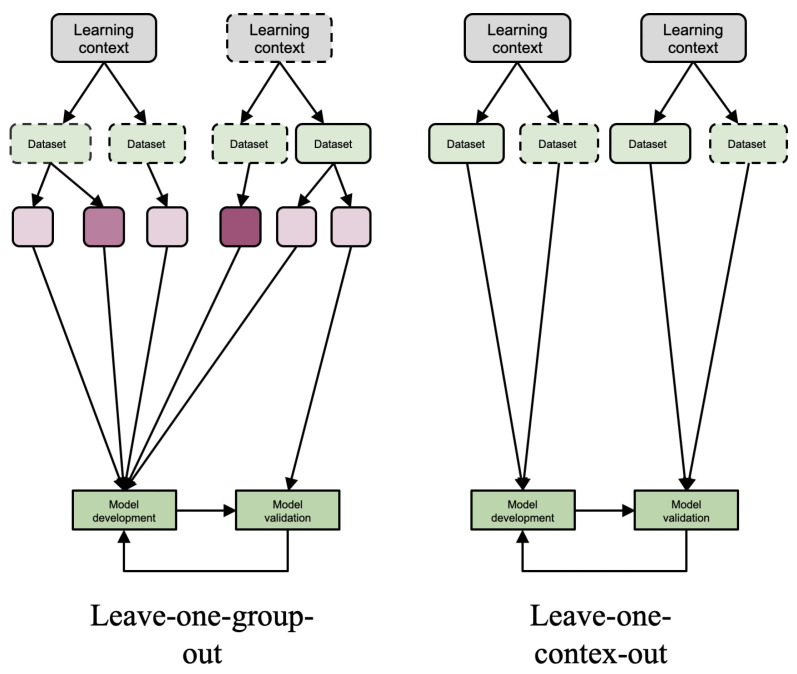
Leave-one group out and leave-one context out evaluation methods.

The EFAR-MMLA assesses generalizability at various levels, starting from the data instance level and moving to the group and context level. The first generalizability level offers an initial performance measure for the model’s expected performance. The next two levels assess the model’s generalizability across different groups and contexts in educational settings. The EFAR-MMLA uses the evaluation methods shown in Figure 1 and Figure 3 to assess these various levels of generalizability.

#### 3.1.1. Instance Generalizability

The first generalizability level assesses the ML model regarding its performance on data that arise from the same distribution as training data but are not used for the model development. It builds on conventional methods of ML model evaluation; e.g., train and test, cross-validation. These model evaluations assess the model’s generalizability at the data instance level (e.g., single activity trace) in the same data distribution. Therefore, we termed the first level of generalizability as instance generalizability. Assessing whether a model performs well only on data from the same distribution may be of limited use in a practical situation, but it is still helpful for MMLA researchers to determine their model’s initial performance measure, which could guide the further development of the model.

The EFAR-MMLA suggests using a hold-out evaluation method to assess instance generalizability. The hold-out method demands a large dataset to allow the ML model to reach its full capacity even if some portion of the data (test set) is not available for the model training [10]. In the case of small datasets, which often happens in MMLA, EFAR-MMLA suggests that researchers use a repeated version of hold-out to assess the ML model.

The EFAR-MMLA also suggests the k-fold evaluation methods as they offer an unbiased estimate of performance [23] and have been found to offer a better performance measure compared to hold-out and bootstrap methods [35]. The EFAR-MMLA suggests a stratified k-fold following Kohavi’s recommendation [35]. The EFAR-MMLA also uses a non-stratified k-fold, which partitions the dataset in random folds with unbalanced data distribution. It simulates a real-world scenario where the model is often expected to perform on data that may be different from its training data. Following Kohavi’s recommendation [35], the EFAR-MMLA suggests 10-folds (k = 10).

The availability of multiple evaluation methods for instance generalizability allows researchers to select the method that is appropriate for their case. For example, the EFAR-MMLA suggests that researchers use hold-out over k-fold cross-validation when the dataset is large and includes a range of cases on the learning constructs under investigation. Considering a researcher with a dataset regarding emotional engagement—collected from a wide variety of participants and settings—that has an approximately equal proportion of labels, the researcher can apply hold-out while evaluating the model. On the contrary, if the dataset size is small, the EFAR-MMLA suggests using k-fold cross-validation to avoid the overfitting which may happen with the hold-out evaluation method. Moreover, using hold-out with a small dataset can give an unreliable estimate of performance depending on the way the dataset is split into training and test sets. In the case of unequal label proportion, EFAR-MMLA suggests using stratified k-fold for model evaluation. For example, if a researcher is building an engagement classifier over data with 80% instances of high and 20% instances of low engagement classes, using a stratified k-fold can offer more reliable performance estimates than the non-stratified k-fold.

The majority of MMLA researchers [20,21,22,26,28,29] have evaluated their models for instance generalizability level while employing various ML models; e.g., SVM, AdaBoost, random forest, neural network and naive Bayes. Among these, random forest is frequently found to be a better model by researchers [13,17,25]. Researchers have mostly used k-fold for their model evaluation. Few researchers, however, have also used a leave-one out strategy for the evaluation of their model’s instance generalizability [21,29]. A model with instance generalizability performs as expected on data from the same learning situation (e.g., same learning activity, same teacher, same learning space, same data collection, etc.) and with the same participants (e.g., the same group composition if collaborative learning). However, changing any of these aspects may cause a degradation in the model’s performance. Thus, a model with instance generalizability has limited applicability in MMLA (in terms of future predictions). Nevertheless, it allows researchers to find ML models that optimally fit the available data. Therefore, evaluation at this level can be used when a researcher’s goal in developing an ML model is to identify predictive features for learning constructs. In fact, MMLA researchers are often interested in identifying the relationship between multimodal data and learning, which can be achieved by evaluating the models on the instance generalizability in the EFAR-MMLA. Still, in such cases, evaluation at higher generalizability levels allows researchers to validate the identified link between multimodal data and learning at stricter levels and also identify biases in the model, as research supports the claim that ML models can discriminate [36] over aspects (e.g., gender, skin-type) while making the prediction.

#### 3.1.2. Group Generalizability

Group generalizability is achieved when the model performs as expected across different student groups. The leave-one group out evaluation method (Figure 3) is used to assess group generalizability [24,37]. In this method, available datasets are partitioned using information from learning contexts (e.g., student’s performance, ethnicity, demographics, etc.). The data from one group are used for model evaluation, while data from other groups are used for model development. This process is then repeated until data from each group are taken for evaluation.

The group generalizability level ideally requires equal distribution of learning labels across various groups (e.g., approximately equal distribution of high/low engagement across different ethnic groups when estimating engagement) in the dataset. However, MMLA researchers do not have this luxury and are often faced with a dataset with unequal data distribution across groups. The models developed on such a dataset are inherently biased. To deal with this issue, the EFAR-MMLA suggests the use of resampling techniques to balance the dataset in terms of the proportion of learning labels across different groups (e.g., male/female, different ethnic groups). This resampling can be done by undersampling, oversampling or hybrid approaches [38]. These techniques employ different strategies to balance the dataset across groups. For example, undersampling reduces the majority group to balance it with minority groups. In contrast, oversampling does the opposite, increasing the data sample in the minority group either by duplication or synthetic data generation (e.g., SMOTE [39]).

The group generalizability level helps MMLA researchers to see their model’s performance across different groups (e.g., gender, demographics), thus allowing deeper model evaluation to identify whether a model is biased to a particular group or not. Such evaluation can also help MMLA researchers to identify the differences in the model’s performance across different student groups, which can further guide the potential development of fair ML models [37]. There is a possibility that the developed model favors a particular group of students to predict learning constructs over others (due to ethnic differences). Such biases should be taken into consideration while evaluating ML models in MMLA, and group generalizability can be helpful for that purpose, as illustrated by [19]. For instance, an MMLA researcher investigating students’ emotional engagement using video and audio data (e.g., facial expression, verbal or non-verbal audio features) is interested in the model’s performance on a dataset that is imbalanced in terms of demographics or gender aspects. Assessing the model’s performance on various student groups according to demographics or gender will help the researcher to identify the biases in the model’s performance before putting the model into real-world practice.

The idea of human-centric model analysis across various groups (based on various characteristics; e.g., gender, race) is not unique to MMLA fields. Other fields—e.g., medical imaging [40], public health [41], or computer vision [36]—have also used such analyses using grouping factors from their datasets while assessing their ML models. Moreover, the UNESCO reports [42,43] “I’d blush If I could: closing gender divides in digital skills through education” and “Artificial intelligence and Gender inequality” emphasize the gender biases coded in AI algorithms and demand the consideration of these during AI development. Furthermore, a model’s bias is not limited to groups across single characteristics (e.g., gender) but also across the interaction of those characteristics (e.g., gender and demographic), giving rise to intersectional AI [44]. For example, recent ML work [36] into the intersectional analysis of ML models in face detection found a bias in face detection algorithms towards dark-skinned women. The assessment of ML models on different grouping factors, which can be determined in light of the purpose behind the development of the model, can help us to mitigate such biases.

#### 3.1.3. Context Generalizability

This level of generalizability is highest in the MMLA studies, and the EFAR-MMLA suggests the leave-one context out evaluation method (Figure 3) to assess it. This method is built upon validity generalization methodology [23]. This methodology works similarly to cross-validation but uses a sample from a different study (or data distribution) to estimate the model’s predictive effectiveness. Thus, leave-one context out requires the collection of additional datasets from a different learning context than that from which data were collected for the model’s development. There is a myriad of dimensions of learning context—from macro (institutional level) and meso (classroom involving teacher, participants) to micro (learning content)—that could be considered for contextual variation. The macro-level dimension brings a significant variation in the context, while micro-level dimensions cause minor contextual variation. For example, some changes that could be taken into consideration to choose a different context include different participants, different learning spaces, different types of learning activities, different learning content, etc. Context generalizability accounts for these all contextual changes from major to minor. Thus, the EFAR-MMLA suggests that researchers explicitly state the dimension on which models have achieved context generalizability.

An ML model with this generalizability performs as expected on data from different learning contexts. For instance, an MMLA model built to assess students’ engagement in a classroom is expected to perform well in the same classroom with the same learning activities but with different students. This desired generalizability can be assessed by evaluating models on context generalizability. Few MMLA researchers have evaluated their models at this level [5,17]. Although it is the most desirable generalizability, greater effort is required to achieve it. Therefore, to reduce the required effort, the EFAR-MMLA recommends that MMLA researchers decide in advance the aspect of the learning context for which generalizability is desired.

The EFAR-MMLA also suggests using the ML model’s performance at the context generalizability level for model comparison and selection purposes. Context generalizability is based on the “validity generalization criterion” [23,31] recommended for model comparison. This criterion can be used to “compare any set of models; they may even differ in terms of the number of parameters” [31] instead of the cross-validation methods that are useful when the dataset is small. In the case of a large dataset, cross-validation tends to pick complex models [45] and may introduce over-fitting. The use of validity criteria avoids the selection of models that achieved better performance simply because of their dependence on a high number of parameters.

### 3.2. Performance Reporting

The EFAR-MMLA suggests reporting a model’s performance at various levels of generalizability with a variation measure to offer information regarding the stability of the model’s performance. Besides, the EFAR-MMLA also suggests the use of two frames of reference as lower and upper bounds of the model’s performance to offer information on the practical value of the model.

#### 3.2.1. Performance Variation Measure

Recent works in ML [46,47] emphasize the importance of how the ML model’s performance is reported. These works suggest including performance variation (e.g., standard deviation, variance, or confidence interval) and hyper-parameter search strategies while reporting. Following these recommendations, the EFAR-MMLA suggests reporting variation along with the average performance measure and hyper-parameter search strategy. The evaluation methods used in the EFAR-MMLA provide multiple performance measures. For instance, in repeated hold-out, a performance measure is obtained in each iteration, and the same happens in k-fold cross-validation. These measures can be used to compute the average performance and standard deviation as the variation across different folds/iterations.

#### 3.2.2. Frames of Reference

MMLA researchers have used different frames of reference (e.g., majority classifier [21], random classifier [22], proportion classifier [19]) in their reporting. Except for the random classifier, all these frames of reference require some knowledge from the collected dataset; e.g., the majority or proportion of class labels. In addition, a model that is worse than random is useless, but being above that level does not make a model useful automatically, especially not in terms of its real practical value. Therefore, we decided to use a lower bound (or baseline) performance that does not require any such advanced knowledge (e.g., class label distributions) of future datasets and offers a better understanding than a random model. The EFAR-MMLA uses the theoretical average model’s performance or the best-known performance of the ML model on the past data. A theoretical average model is a fixed strategy model that estimates a single value for all the cases (for regression problem) or estimates class labels with equal probability (for classification problem). For example, a theoretical average model will always estimate a test performance (that ranges from zero to 100) of 50 for all cases.

To better estimate how far a model is from achieving its expected performance, the EFAR-MMLA uses an upper bound for the model’s performance. The learning constructs in MMLA studies are often complex and require human experts to annotate the dataset for predictive modeling. The EFAR-MMLA uses these annotations as a means to compute the human-level agreement and use it as an expected performance level for the developed ML model.

**Figure 4:**
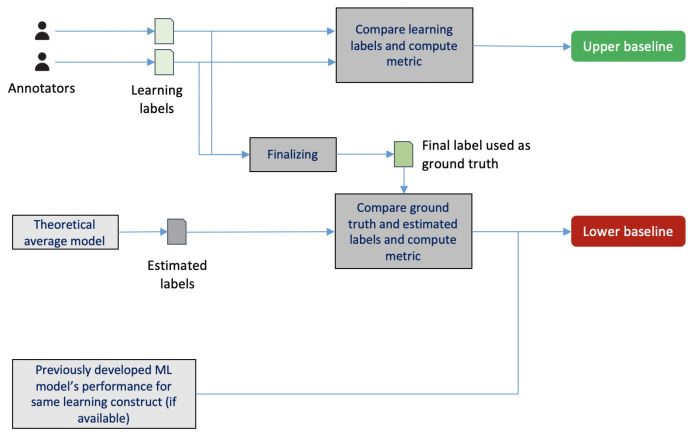
Process to compute frames of reference in EFAR-MMLA.

Figure 4 depicts the process to compute the upper and lower bound of performance to be used in reporting. To compute the upper bound, the learning labels obtained from annotators are taken, the performance metric (e.g., root mean square error or kappa) is computed between those labels, and the computed metric is used as the upper bound. To compute the lower bound, first, the finalized learning labels or ground truth are taken; then, the average of the theoretical minimum and maximum value of the learning construct under analysis is computed and assigned to each instance in the dataset. These assigned labels are then compared with the ground-truth to compute the performance metric. In the case of a classification problem, class labels with equal probability are assigned to the instances of the dataset, and then these labels are compared with the ground truth to compute the performance metric. The obtained performance metric is used as the lower bound. In case an ML model has already been developed on the past data for the same learning construct under investigation, then that model’s performance is used as a lower bound.

We present a working example to illustrate the computation process. Consider two annotators (A and B) have labeled student group behavior at the SMU (sustaining mutual understanding) dimension of collaboration quality. The annotators used a 60 s time window to code a 10 min group activity, giving 10 codes. The annotations are in the range of −2 (very poor) to +2 (very good). Table 2 shows an example of the assigned codes.

Once we have annotated labels, we compute the performance metric used in the analysis (e.g., RMSE in the case of regression). The following equation shows the formula for RMSE and how it is used to compute the upper bound frame of reference (which is 1.04 for this example).

(1) $$RMSE\left(A,B\right)=\sqrt{\frac{1}{N}\sum_{n=1}^{N}{\left(a_{n}-b_{n}\right)}^{2}}$$

where $a_{n}$ and $b_{n}$ refer to the labels assigned to frame *n* by annotator *A* and *B*, respectively. *N* refers to total number of frames.

To compute the lower bound, we need labels finalized by both annotators. We use a theoretical model here that estimates an average for all the frames. As the range of assigned codes is −2 to +2, a theoretical model will always assign a label of zero (average of −2 and +2) to each frame. Table 3 shows the final labels and the one estimated by the theoretical model. We now apply the RMSE formula to the final and theoretical model’s labels; this gives us 1.37, which can be used as a lower bound frame of reference.

### 3.3. Current State of MMLA Research from EFAR-MMLA Point of View

Table 4 provides an overview of existing MMLA works using ML from the EFAR-MMLA point of view. This initial evidence shows that the majority of the works mentioned evaluated their models at the first EFAR-MMLA generalizability level (instance generalizability), possibly due to the unavailability of an additional dataset from a different context. This highlights the need for evaluation at higher generalizability levels to build practice-ready ML models in MMLA.

Considering performance reporting, we can observe an inconsistency in how researchers report their model’s performance (Table 4). Moreover, the variation in the developed model’s performance and the approach employed to tune the models are rarely reported. Few researchers (Martinez-Maldonado et al. [24]) have supplemented their reporting of performance variation with an explanation; e.g., variation as an indication of oscillation in a model’s predictive ability. We also noticed the use of different frames of reference by researchers and inconsistent use of terms. For example, some researchers [21,26,27] have used a majority baseline to compare their results. This baseline is computed by assigning each data instance a class label that occurs most often in the dataset and then computing the performance metric with these assigned labels and ground truth. Some researchers have referred to this as a “chance baseline” [21,27] or “no-information system performance” (a system that predicts without using any information from the dataset) [26]. This usage of terms conflicts with a random baseline (which is often considered as a chance baseline). Moreover, the majority baseline needs information from the dataset (the proportion of the class labels), in that sense, it is not truly a no-information system. Furthermore, the prevalence of different baselines with conflicting use of terms in the field confuses new MMLA researchers when deciding on a baseline for their research work.

## 4. Illustrative Case Study

To illustrate how the framework could be applied to a real MMLA research effort and the added value that it may bring, we present a case study in which different ML models were developed to estimate the collaboration quality and its sub-dimensions. In this study, two datasets were collected from two learning contexts that were different in terms of learning activity and the student group’s composition.

### 4.1. Motivation and Context of the MMLA Project

Despite the benefits of collaborative learning [48], it is well known that students need scaffolding to collaborate effectively. While teachers can provide this guidance during the learning process, it is extremely demanding to monitor and support several groups at the same time [49], especially when the learning activity occurs across physical and digital spaces. MMLA researchers have built ML models to estimate collaboration behavior to support teachers [20,24,50]. The majority of this research has employed classification models, even though collaboration quality is arguably a continuous spectrum [20,24]. These models from existing research can identify clear cases of high/low collaboration quality but may fail to reliably detect intermediate cases. Indeed, Martinez-Maldonado et al. [49] have also mentioned this gap, identifying the need for more nuanced models. The illustrative case study depicts our ongoing work in addressing this challenge of estimating collaboration quality and its sub-dimensions along a continuous spectrum/scale.

The study was conducted in an upper-secondary school classroom with 10 students during a Biology course in the autumn of 2019. Two sessions were conducted in the same classroom with the same teacher, students, learning space and data gathering techniques but different group compositions and problem topics (cell respiration and genetically modified organism). The learning activity for both sessions was designed ahead of the study by an educational sciences researcher, in collaboration with the teacher. The designed activities had two parts: (1) a lecture by the teacher on the problem topic, and (2) students working in groups on the given problem (Problem A: discussing the questions on cell respiration and writing their answers in Etherpad –An open-source collaborative text editor–; Problem B: discussing the ethical concerns of growing genetically modified crops and reporting them in Etherpad). At the beginning of the second part of the activity, researchers provided information about the aim of the study and data collection. Then, written consent for data gathering was collected from students. Both activities were completed along comparable time spans (30–35 min). The audio of the group conversations and the log data from the collaborative writing were collected during the activity. Table 5 presents the information from both sessions.

### 4.2. Research Problem

The estimation of collaboration on a continuous scale (rather than a few categories) in face-to-face classroom settings is a challenging problem. The ML models built in MMLA research to estimate discrete levels of collaboration quality also fail to offer additional information on the underlying reasons for the estimated quality level, thus offering fewer clues about how to scaffold students when collaborating (i.e., the actionability of the MMLA estimations is limited). This case study employs regression models to estimate collaboration quality on a continuous scale, and it also attempts to estimate various collaboration quality sub-dimensions [51] (argumentation, collaboration flow, knowledge exchange, sustaining mutual understanding, cooperative orientation, structuring problem-solving process and time management, individual task orientation) to offer more actionable estimation results.

### 4.3. Methods

#### 4.3.1. Data Gathering

**Figure 5:**
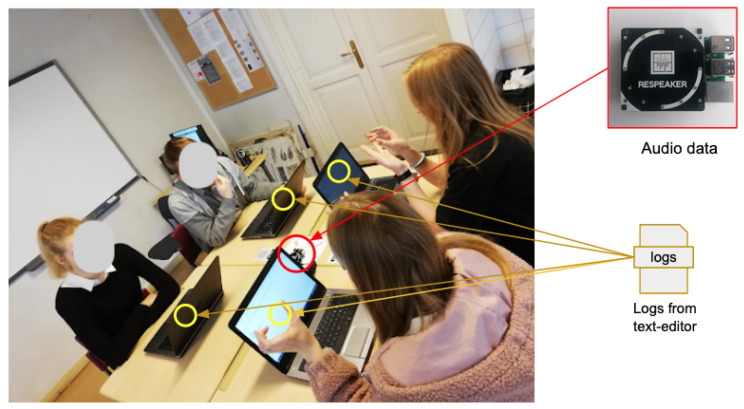
Learning context.

We used a prototype—CoTrack—based on Raspberry Pi (model 3B+) with a ReSpeaker (https://wiki.seeedstudio.com/ReSpeaker_4_Mic_Array_for_Raspberry_Pi/, accessed on 17 February 2020) microphone array (with four microphones) to capture the audio data. Our prototype [52] used a VAD (Voice Activity Detection) algorithm to detect voice activity every 20 ms and a DoA (Direction of Arrival) algorithm to detect the sound’s incoming direction every 200 ms. The prototype sent the detected direction, along with the timestamp and group label to the server using MQTT (Message Queuing Telemetry Transport) protocol [53]. We also used Etherpad to allow students to share information and prepare a consolidated solution to the given problem. We used the Network Time Protocol (NTP) for time synchronization between Etherpad and Raspberry Pi prototypes. All collected data were stored on the server in the form of CSV files. Figure 5 shows the data collection setup during the learning activity.

#### 4.3.2. Data Processing

We decided to use a 30 s time window in data processing to align and summarize the different data sources and to provide human-labeled ground truth for the collaboration quality and its sub-dimensions. This window was chosen based on recommendations from previous research [24] (which did not find significant performance improvements for 60 or 90 s time windows over 30 s windows). We obtained 325 data instances after summarizing with a 30 s window. We extracted the following features from collected audio and log data.

**Simple Features**
  Weinberger and Fisher [54] highlighted the amount of participation as one of the key quantitative measures in collaborative learning, and this is considered a useful indicator for collaborative behavior [24]. In our case study, we computed the amount of participation in physical and digital spaces in terms of speaking time, turn-taking and writing activity in Etherpad (please refer to Table 6). To extract speaking time and turn-taking, the direction of audio captured with the microphone array was mapped to each student according to their sitting position around the prototype. This mapping provided us with the sequence of speaking turns taken by students. We counted the total number of turns taken by each student in a group for each 30 s window, as well as their total speaking time (in 200 ms increments, which was the granularity of the audio direction detection algorithm). From the Etherpad logs, we obtained the number of characters added or deleted by each student. These features were first collected at the individual level. We then used PCA (Principal Component Analysis)-based fusion to obtain group-level features from individual features [55]. PCA is a dimensionality reduction technique that reduces the number of attributes in a dataset while preserving most of the variance in the data. Our preliminary analyses showed PCA to be a better-performing fusion method than average and entropy-based methods for individual student data fusion (see [56]).
**Acoustic Features**
  We also extracted acoustic features (e.g., pitch, fundamental frequency, energy) from the group audio data of all the collaborating students. This decision was based on previous collaboration modeling research [13,27,57], which achieved higher classification accuracy compared to other types of features in laboratory settings [13]. We used the OpenSmile toolkit (https://www.audeering.com/opensmile/, accessed on 12 August 2020) and extracted 1584 different acoustic features (please refer to Appendix A Table A1 for a full list). Given the high dimensionality of these audio features (e.g., more features than the total number of data points), we used several dimensionality reduction strategies on this feature set. First, removing highly-correlated features (with a correlation > 0.90) left us with 803 features; we then applied PCA dimensionality reduction for further reduction, resulting in 156 features explaining 90% of the variance in the data.
**Linguistic Features**
  We used a speech-to-text service (Otter.ai: https://otter.ai/, accessed on 16 May 2020) to obtain transcripts of the recorded audio automatically. We decided to use this approach instead of manually transcribing audio because of its easier integration in the automation of the application to estimate collaboration quality. We extracted linguistic features (e.g., frequency of “we”, “you”, “our” ) from the transcript for each group. The extracted features were based on previous research that has found differences between collaborative and non-collaborative behaviors in a group’s usage of first/second person singular pronouns (I, you), first-person plural pronouns (we, us) [58] and the numbers of words and sentences [25]. We extracted the number of times these words were used for every 30 s window in addition to the total number of words and the number of “wh” words (e.g., what, why, where).

#### 4.3.3. Data Annotation

**Figure 6:**
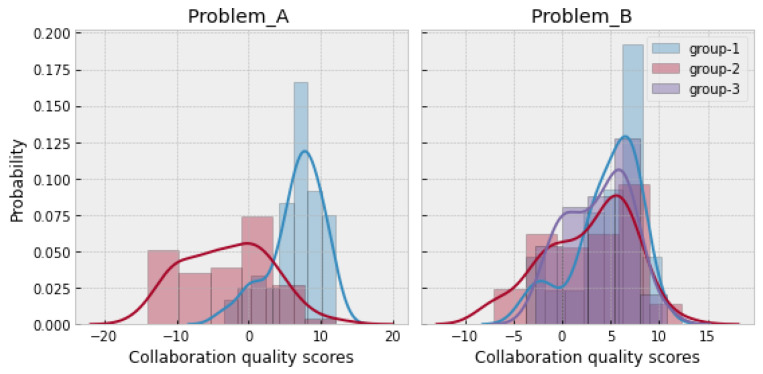
Distribution of collaboration quality scores.

To obtain a ground-truth measure of collaboration quality, our research team manually labeled the videos from each group of collaborating students. We used the rating handbook created by Rummel et al. [51] (which is an adaptable version of Meier et al.’s rating scheme [59]) to quantify the groups’ collaboration quality for each 30 s window. This rating scheme involves seven dimensions of collaboration quality: sustaining mutual understanding (SMU), knowledge exchange (KE), argumentation (ARG), collaboration flow (CF), cooperative orientation (CO), structuring problem-solving process and time management (SPST) and individual task orientation (ITO). Each dimension is given a score between −2 to +2. The rating scheme codes the first six dimensions at the group level, while the last dimension (ITO) is rated at an individual level. We used the average of ITO as a group-level measure of task orientation. We then added all these dimensions’ scores to obtain an overall collaboration quality score between −14 to +14. Figure 6 shows the distribution of collaboration quality in the $Problem_{A}$ and $Problem_{B}$ datasets. In the case of $Problem_{A}$, group 1 showed higher collaboration quality scores, while group 2 showed low collaboration quality scores. In the case of $Problem_{B}$, all three groups showed an approximately similar distribution of collaboration quality scores.

Two researchers used the rating handbook [51] as training material and independently coded 10 min of video from collaboration activities. After the first iteration, Cohen’s kappa was computed and found to be below an acceptable level (kappa < 0.60 for all dimensions). Both researchers discussed the disagreement in their assigned codes and reached a consensus, leading to a refined version of the coding handbook. This process was repeated two more times (both times, the Cohen’s kappa score was still low) and further revisions were made to the handbook. The final, updated rating handbook was used to code the entire collaborative activity. In this fourth iteration, a substantial agreement (0.80 > kappa > 0.61) based on [60] guidelines was achieved for all seven dimensions (Table 7).

### 4.4. Analysis

We employed five different regression models (K-nearest neighbors, random forest, ADA boost, gradient boost, and neural networks) with various feature sets (simple features, acoustic features, linguistic features, all features) in our attempt to estimate eight labels (overall collaboration quality and its seven sub-dimensions). Thus, we had a total of 160 (5 × 4 × 8) regression models that were evaluated using the five evaluation methods (repeated hold-out, stratified k-fold, k-fold, leave-one group out, leave-one context out) proposed in our evaluation framework. We decided to use multiple evaluation methods for instance generalizability to see how performance varied when used with repeated hold-out, stratified k-fold and non-stratified k-fold approaches. We used a grid-search strategy to tune hyper-parameters (analysis code is available here: https://github.com/pankajchejara23/Sensors_EFARMMLA_Codes, accessed on 20 March 2021).

### 4.5. Results

We selected root mean square error (RMSE) as the main performance measure, following the recommendation from Chai et al. [61]. We computed the RMSE for a no-information predictor that always outputs the theoretical average (i.e., zero) for each sub-dimension and overall collaboration quality scores (as a lower bound for the performance of our models). We also computed the upper-bound frame of reference by applying the RMSE formula (Equation (1)) on the annotated labels obtained from annotators. As suggested by our evaluation framework, these frames of reference helped us to assess the practical applicability of our models when estimating collaboration quality and each of its sub-dimensions (Table 8). We used the evaluation methods from the proposed framework to assess the developed regression models (repeated hold-out and k-fold for instance generalizability, leave-one group out for group generalizability and leave-one context out for context generalizability); refer to Table A2, Table A3, Table A4, Table A5 and Table A6 for the RMSE metrics for all employed regression models with different feature sets at different generalizability levels.

**Figure 7:**
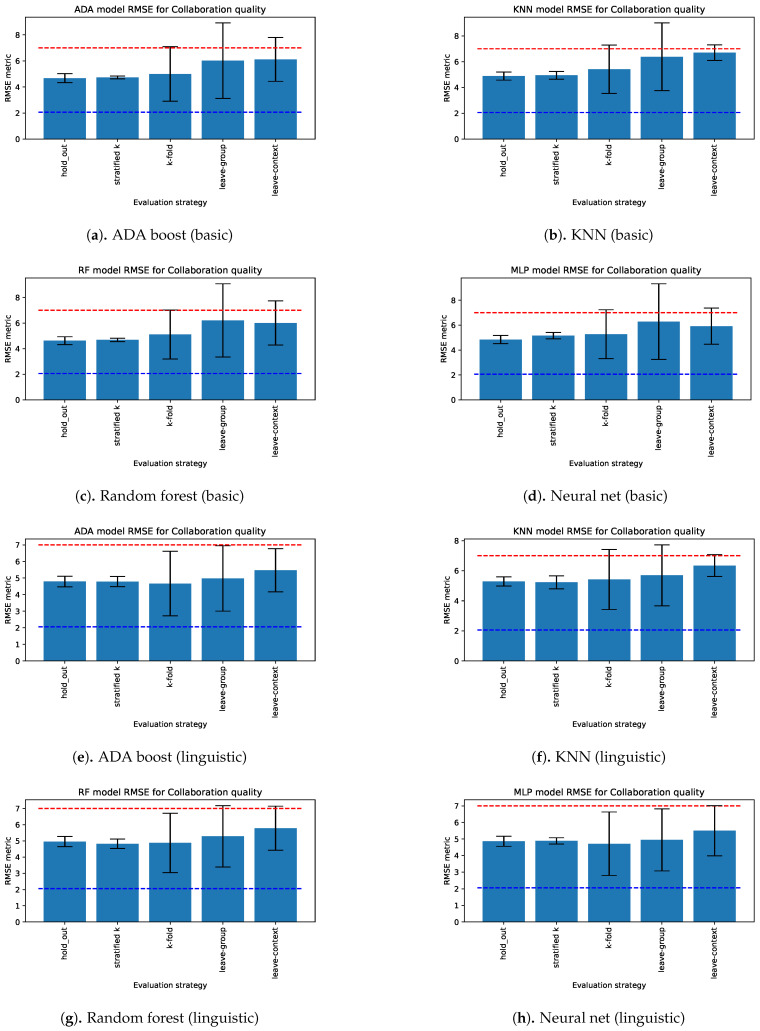
Evaluation of regression models estimating collaboration quality using basic and linguistic features in terms of RMSE (smaller is better). The red dashed line represents the theoretical average (i.e., no-information) predictor’s performance, and the blue dashed line represents the human performance level.

We used the framework’s guidelines to evaluate and report on the model’s performance at different levels of generalizability. Figure 7 shows the generalizability evaluation of ML regression models using basic and linguistic features with frames of reference from EFAR-MMLA. We chose these features over acoustic and all features due to the high variance in the models’ performance when using acoustic features and the small or lack of improvement in performance when using all features. Therefore, for illustration purposes, we show here only examples of models using basic and linguistic features.

The regression models (Ada boost, random forest, KNN, neural net) with basic and linguistic features estimated collaboration quality better than the theoretical average model’s performance (Figure 7) for all generalizability levels using repeated hold-out, k-fold with and without stratification, leave-one group out and leave-one context out. However, considering the standard deviation, we can see that these models showed high variation in their RMSE, indicating their instability in performing consistently with k-fold, leave-one group out and leave-one context out approaches. The high variation on k-fold shows that, in some of the evaluations, the model performed close to (or worse than) the lower-bound reference frame while it performed close to a human level with some of them. The models were only able to achieve the first level of generalizability (i.e., instance generalizability), as shown by the low RMSE and low standard deviation, with repeated hold-out and stratified k-fold evaluation approaches. The higher variation in performance using k-fold, leave-one group out and leave-one context out approaches suggests the need for more datasets to further train the models and improve their performance at higher levels of generalizability.

**Figure 8:**
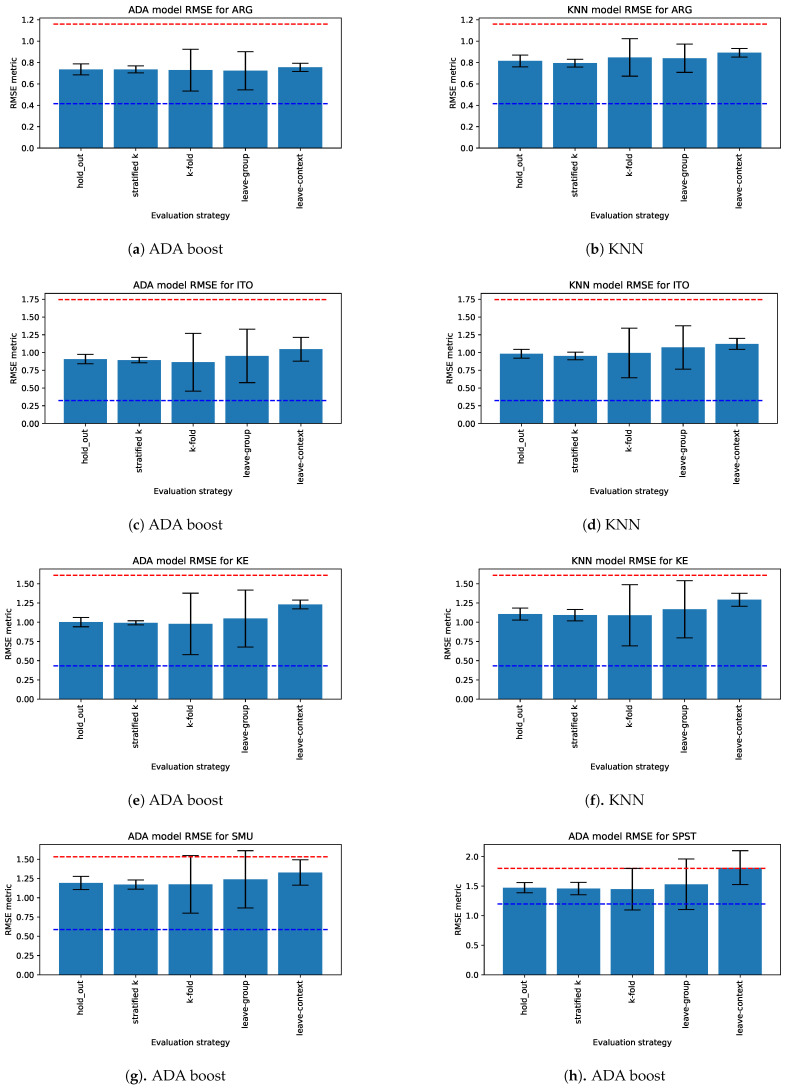
RMSE of regression models estimating the dimension of collaboration using linguistic features.

The regression models also showed better performance in estimating the ARG, KE and ITO dimensions of collaboration quality than the lower bound on all generalizability levels. For example, Figure 8a–f shows the RMSE of Ada boost and the KNN regression model using linguistic features for those dimensions. These models showed a comparatively higher variation with K-fold and leave-one group out approaches than with other evaluation methods. Although the variance was higher when evaluating group generalizability using leave-one group out, the performance was still better than the lower bound reference frame (even the worst model performed better with leave-one group out). The employed regression models performed poorly in estimating other dimensions (SPST, CF, CO, SMU). For example, the Ada boost model for the SPST dimension performed poorly with k-fold, leave-one group out and leave-one context out approaches (Figure 8h).

## 5. Discussion

MMLA researchers have used a wide variety of methods to assess and report on the generalizability of ML models. In MMLA, this requires the consideration of generalizability levels that are relevant to the educational context while assessing the model. Moreover, the lack of a standard for ML model evaluation in MMLA makes it difficult to compare ML proposals and to understand the progress that this field is making towards practically relevant solutions. Our framework improves this situation by making the following contributions.

- Assessing models at different generalizability levels
  Evaluating ML models at different generalizability levels helps researchers to see how their model’s performance varies when moving towards stricter generalizability levels. In our case study, we show how the ML regression models performed better than the lower (no-information) frame of reference in estimating overall collaboration quality and some of its sub-dimensions (e.g., ITO, ARG), when evaluated using train and test evaluation or stratified k-fold corresponding to the instance generalizability. However, the performance degraded substantially when models were evaluated using more stringent assessments in the EFAR-MMLA. The most likely reasons for this performance degradation are the small dataset size and the small number of contexts/groups from which data were gathered. The assessment at different generalizability levels (group and context) helped us to clearly see that the model is not generalizable enough to make it useful in practice.
- Understanding the rationale for performance variation
  The EFAR-MMLA can help us to understand the reason for performance variations by looking systematically and evaluating ML models at different generalizability levels. In our case study, we found significant variations in the performance of our ML models at the instance and group generalizability levels. These variations led us to further explore the underlying reason by looking into the various cross-validation units (e.g., student groups). In our case, we identified a group that was actively participating (in terms of speaking time and characters added or deleted) but mostly with off-topic discussion. This led to human raters scoring this group lower in collaboration quality, but this was undetected by our models (given the types of features that we included in the modeling), thus leading to poor model performance. This illustrates that our models were not able to generalize to that group’s behavior. It also made us consider the inclusion of additional features (e.g., content-based) in a future version of our ML models that could help in mitigating the identified issue.
- Offering better comprehensibility regarding the model’s performance
  The other benefit of the proposed framework is the increased understanding of the performance reporting, both for the research team doing the reporting and for readers of that report. Although the mean performance of our model in terms of instance generalizability (k-fold evaluation) was better than the no-information lower bound, its high variation suggested that the performance was not stable (i.e., it was likely to fail on future data). The inclusion of a performance upper bound allowed us to see the extent to which the model deviated from its expected performance.
- Bringing another perspective of bias identification
  Generalizability is a highly sought-after characteristic by researchers across domains (e.g., clinical research, computer vision). However, this emphasis is not necessarily needed in every scenario, and it has also been criticized in other fields (e.g., clinical research) [62]. Considering an example similar to [62], but in an educational context, to understand this further, a researcher developed an ML model to estimate engagement in the classroom of primary students. The researcher validated models’ performance in other classrooms with primary students and found it to achieve moderately stable performance. Now, the researcher aims to further improve the model to work with other students (e.g., secondary, higher secondary); the existing model may suffer significantly reduced performance for even primary students to make it generalizable to other students. If the model generalizes well to other students, that is certainly positive; however, if it does not generalize to students other than primary but performs fairly for primary students, it is still useful, and the lack of generalizability to other students does not necessarily undermine its value. In this direction, besides supporting researchers to evaluate their models for generalizability at the group and context level, EFAR-MMLA brings another perspective regarding the identification of the biases in the models when performed with different groups or contexts. Such biases can help researchers and the community to identify the scenarios in which a model is useful and where and how much its performance degrades when changing group and contextual characteristics. This perspective can be useful for the MMLA community to take a further step towards developing practically relevant models for real-world educational settings.

The framework also has an implication in terms of the practicality of ML model assessment. Non-ML researchers often face difficulty regarding the selection of model evaluation methods and their use in their research; this requires researchers to iterate on the process of adapting the model, find the model’s parameters and assess the model, which in itself demands a significant effort from researchers. To simplify this task to some extent, we used the proposed framework as a guide to develop a toolset in Python to automate this process of model development and model evaluation at different levels of generalizability. The tool is openly available (https://github.com/pankajchejara23/EasyRegression, accessed on 20 March 2021) as a starting point that we hope the MMLA community will adopt and expand upon.

The EFAR-MMLA framework can also complement other MMLA conceptual tools, such as the M-DVC (Multimodal Data-Value Chain) [63] and MLeAM (Multimodal Learning Analytics Model) [4], in systematizing the development process of MMLA solutions. The M-DVC tool supports the communication between multiple stakeholders (e.g., developers, researchers) and provides an MMLA development process in an iterative manner. This process includes multiple steps, including analysis with ML model training [63,64]. The EFAR-MMLA can complement this analysis step by including model evaluation at different generalizability levels, which can help researchers to improve their model’s performance and understand its expected performance in different contexts. The MLeAM conceptual tool is proposed to help researchers to gain a common understanding of MMLA development, focusing on the systematization of ML use in MMLA. This tool has a prediction step involving sub-steps; e.g., training the model, validating the generalizability of the models. Our framework elaborates that step with more detailed guidelines on the assessment of generalizability and its reporting in MMLA.

The EFAR-MMLA framework could also be used in more general Learning Analytics (LA) research. The data collected in LA research are often large enough to allow a model to reach its capacity even when some part of the dataset (in the form of test data) is kept for model evaluation. In such cases, hold-out can be preferred over cross-validation methods for model evaluation as “the usefulness of cross-validation is limited to small datasets” [31]. The variation in performance can be computed using the “normal approximation” method [10]. The leave-one group out and leave-one context out evaluation methods can help in assessing generalizability at the group and context level, respectively. In LA, some research work [37] has also proposed the use of a leave-one group out strategy to increase the model’s fairness; i.e., whether the model is performing similarly across different student groups or favoring a particular group. The context generalizability can help LA researchers to assess their model’s performance across different contexts (e.g., change in participants, learning activity or learning space) which can also help in the exploration of the relationship between data and learning constructs across contexts.

The EFAR-MMLA framework, as presented in this paper, is not without limitations. In its current version, the framework only addresses the evaluation of models for *supervised ML* tasks, where there is a target learning construct (or “learning labels” [4]) that needs to be estimated on future data by the ML model in different evaluation schemes. The EFAR-MMLA uses the model’s performance to assess the generalizability at different levels with the help of proposed frames of reference. The framework does not address the same evaluation for unsupervised learning problems because there is a lack of well-established metrics for the performance for such problems. The EFAR-MMLA in its current version does not deal with the issue of model selection. The later expansion of the framework could address offering advice on ML model selection for specific MMLA problems or learning constructs. Besides, the framework currently does not suggest the size of the dataset or the number of datasets (i.e., in terms of how many different contexts they cover) that would suffice to achieve context generalizability. Further empirical tests for different educational constructs would be needed to formulate relevant guidelines. We also like to note the following limitation of our case study: we had access to datasets with small contextual variation (e.g., learning content and group composition), and other dimensions of contextual variation (e.g., change in learning space or participants) could have been explored.

## 6. Conclusions and Future Work

MMLA research based on machine learning is ongoing, and it still requires the development of consistently high-performing models at different generalizability levels to make its way to real-world practice. In an educational context, an ML model’s performance being evaluated in conventional ways (e.g., a train–test split) is not sufficient, because that can only inform us about the model’s generalizability on the data level (e.g., activity trace). Further efforts should be invested into analyzing a model’s performance with different student groups, contexts and classrooms. These more extensive evaluations can offer a better generalizability assessment than simple train–test splits. The EFAR-MMLA offers concrete guidelines on ML model assessment and reporting in the context of MMLA but also opens a discussion among MMLA researchers regarding the assessment and reporting of ML models in the context of education.

The majority of ML-based research in MMLA to date has involved the collection of (often small) datasets, building ML models and conveying their findings to the community in an ad-hoc manner. However, as a community, we need to start looking at the practical relevance of our models. This requires a shift in the focus of researchers from assessing models at a low level (instance generalizability) to higher levels (e.g., group, classroom and school-level generalizability), which is not an easy task. Considering the factors involved in MMLA research (e.g., complexity in data collection, data preprocessing, data cleaning and data analysis), it becomes quite difficult for a researcher or even a research group to collect, build and assess ML models on multiple datasets. In this context, international collaboration among MMLA researchers can ameliorate the problem through the use of joint datasets from diverse educational settings and wider generalizability evaluations.

As the next step in our research, we plan to gather more datasets from different contexts and use the EFAR-MMLA to evaluate and improve our model’s performance at higher generalizability levels. We will also extract additional features from student’s written and spoken text to address the current limitations of our model in dealing with students’ off-topic discussion issues causing performance degradation. More broadly, we expect to expand the EFAR-MMLA by starting discussions within the MMLA research community. This discussion can help the community to accumulate knowledge on the maturity of ML models in a more consistent way and to take further steps towards building MMLA solutions that can be used in authentic educational practice.

## Figures and Tables

**Table 1:** Assessment methods of ML models’ generalizability in MMLA.

Article	Learning Construct	Problem Type	Model	Dataset Size	Evaluation Method
[5]	Cognitive performance	Regression	SVM, GP	1724	Leave-one study out
[25]	Collaboration quality in CL	Classification	RF, SVM, NB	40	5-fold cv
[18]	Skill acquisition	Regression	RF	252	Hold-out
[13]	Collaboration in CL	Classification	RF	325	10-fold cv
[20]	Artefact quality	Regression	NN	18	4-fold cv
[17]	Teaching activity,	Classification	SVM, LSTM	5561	Leave-one session out
	Social level		RNN		
[22]	Collaboration level	Classification	SVM	117	10-fold cv
[26]	Collaboration quality	Classification	Ada	1623	5-fold cv
[27]	Collaboration quality	Classification	SVM, RF	2942	Hold-out
[21]	Type of dialogue in group	Classification	K-means	1443	Leave-one student out
[28]	Presentation skill	Classification	LR	448	10-fold cv
[29]	Agreement	Classification	Ada, SVM, NN	28	Leave-one out
[24]	Collaboration levels in CL	Classification	NB, DT	700	Leave-one group out
					10-fold cv

CL: Collaborative Learning, GP: Gaussian Process, SVM: Support Vector Machine, RF: Random Forest, NN: Neural Network, RNN: Recurrent Neural Network, LSTM: Long-Short Term Memory, Ada: AdaBoost, LR: Logistic Regression, DT: Decision Tree, NB: Naive Bayes, CV: Cross-Validation.

**Table 2:** Example for computing upper bound.

Frame No.	1	2	3	4	5	6	7	8	9	10
A	1	2	−2	2	1	1	1	2	2	−2
B	1	1	−2	0	−2	0	1	2	1	−2

**Table 3:** Example for computing the lower bound.

Frame No.	1	2	3	4	5	6	7	8	9	10
Final	1	2	−2	1	1	1	1	2	2	−2
Theoretical	0	0	0	0	0	0	0	0	0	0

**Table 4:** Current State of MMLA Research from the EFAR-MMLA Point of View.

Article	Generalizability Levels	Performance Reporting		
		Performance Variation	Frame of Reference	HP Tuning
[5]	Context	Std	Random	Not reported
[25]	Instance	Not reported	Random	Grid
[18]	Instance	95% CI	None	Not reported
[13]	Instance	Not reported	None	Not reported
[20]	Instance	Variance	None	Not reported
[17]	Context	IQR	None	Manual and grid
[22]	Instance	Not reported	Random	Not reported
[26]	Instance	Not reported	Majority	Not reported
[27]	Instance	Not reported	Majority	Manual
[21]	Instance	Not reported	Majority	Manual
[28]	Instance	Not reported	None	Not reported
[29]	Instance	Not reported	Random	Not reported
[24]	Group, context	Std	Proportion	Not reported

Std: standard deviation, HP: hyper-parameter, IQR: inter quartile-range, CI: confidence interval.

**Table 5:** Description of the learning scenarios in which the datasets were collected.

Dataset	Group Number	Group Size	Data Sources	Problem Topic
Problem_A	2	4	Audio and log	Cell respiration
Problem_B	3	3–4	Audio and log	Ethical codes on growing GMO

**Table 6:** Simple feature set (all features calculated for every 30 s window).

Feature	Description
Speaking time	Speaking time in seconds for each student
Turn-taking	Number of speaking turns taken by each student
Char-add	Number of characters added in Etherpad by each student
Char-del	Number of character deleted in Etherpad by each student

**Table 7:** Inter-rater agreement of human coders in each collaboration quality sub-dimension (Cohen’s kappa).

SMU	CF	KE	ARG	SPST	CO	ITO-1	ITO-2	ITO-3	ITO-4
0.71	0.91	0.74	0.80	0.65	0.68	0.72	0.76	0.75	0.78

**Table 8:** Frames of reference for collaboration quality and its sub-dimensions in RMSE.

Frame of Reference	CQ	SMU	CF	KE	ARG	SPST	CO	ITO
Predictor using theoretical average (lower bound)	7.00	1.53	1.26	1.61	1.15	1.79	1.15	1.74
Human performance (upper bound)	2.06	0.59	0.33	0.43	0.41	1.20	0.58	0.32

## Data Availability

The data presented in this study are openly available in FigShare at https://doi.org/10.6084/m9.figshare.14444351.v1, accessed on 20 March 2021.

## Abbreviations

ML	Machine learning
MMLA	Multimodal learning analytics
EFAR-MMLA	Evaluation framework to assess and report generalizability of ML models in MMLA
CL	Collaborative learning
CV	Cross-validation
CQ	Collaboration quality
ARG	Argumentation
KE	Knowledge exchange
CO	Cooperative orientation
SPST	Structuring problem-solving process and time management
SMU	Sustaining mutual understanding
ITO	Individual task orientation
CF	Collaboration flow
GP	Gaussian process
SVM	Support vector machine
RF	Random forest
NN	Neural network
RNN	Recurrent neural network
LSTM	Long short-term Memory
Ada	Ada boost
LR	Logistic Regression
DT	Decision tree
STD	Standard deviation
HP	Hyper-parameter
IQR	Inter-quartile range
CI	Confidence interval
NB	Naive Bayes

## Appendix A

**Table A1:** Acoustric features from OpenSmile Tool.

Feature Name	Number of Features
MFCC	630 (15 Fe, 15 D, 21 F)
Mel frequency	336 (8 Fe, 8 D, 21 F)
Linear spectral coefficient	336 (8 Fe, 8 D, 21 F)
Loudness	42 (1 Fe, 1 D, 21 F)
Voicing	42 (1 Fe, 1 D, 21 F)
Fundamental frequency envelope	42 (1 Fe, 1 D, 21 F)
Jitter	38 (1 Fe, 1 D, 19 F)
Jitter(DP)	38 (1 Fe, 1 D, 19 F)
Shimmer	38 (1 Fe, 1 D, 19 F)
Pitch onsets	38 (1 Fe, 1 D, 19 F)
Duration	38 (1 Fe, 1 D, 19 F)

## Appendix B

This section presents the RMSE performance of regression models in estimating collaboration quality and its sub-dimensions using audio and logs data in classroom settings.

**Table A2:** RMSE of k nearest neighbor regression model.

Strategy	Features	CQ	ARG	CF	CO	ITO	KE	SMU	SPST
RHO	Basic	4.88 (0.10)	0.83 (0.00)	1.23 (0.01)	0.98 (0.00)	0.87 (0.01)	1.06 (0.01)	1.19 (0.01)	1.51 (0.01)
	Acoustic	5.81 (0.21)	0.90 (0.00)	1.42 (0.01)	0.99 (0.00)	0.96 (0.01)	1.23 (0.01)	1.34 (0.01)	1.60 (0.01)
	Linguistic	5.29 (0.09)	0.82 (0.00)	1.34 (0.01)	0.98 (0.00)	0.98 (0.00)	1.11 (0.01)	1.32 (0.01)	1.60 (0.01)
	All	5.65 (0.25)	0.90 (0.01)	1.42 (0.01)	0.97 (0.00)	0.96 (0.01)	1.17 (0.01)	1.32 (0.01)	1.59 (0.01)
SKF	Basic	4.94 (0.09)	0.80 (0.00)	1.20 (0.00)	0.99 (0.00)	0.86 (0.01)	1.03 (0.01)	1.22 (0.01)	1.49 (0.00)
	Acoustic	5.82 (0.07)	0.90 (0.00)	1.44 (0.00)	0.99 (0.00)	0.99 (0.00)	1.21 (0.01)	1.33 (0.01)	1.61 (0.01)
	Linguistic	5.23 (0.19)	0.79 (0.00)	1.37 (0.01)	0.99 (0.00)	0.95 (0.00)	1.09 (0.01)	1.29 (0.01)	1.62 (0.00)
	All	5.83 (0.07)	0.91 (0.00)	1.41 (0.01)	0.96 (0.00)	0.95 (0.00)	1.17 (0.00)	1.34 (0.00)	1.58 (0.00)
KF	Basic	5.42 (3.51)	0.85 (0.06)	1.36 (0.12)	0.99 (0.07)	0.96 (0.17)	1.13 (0.15)	1.31 (0.17)	1.53 (0.16)
	Acoustic	5.34 (6.02)	0.90 (0.04)	1.34 (0.25)	0.95 (0.07)	0.91 (0.19)	1.15 (0.26)	1.27 (0.22)	1.68 (0.08)
	Linguistic	5.42 (4.00)	0.85 (0.03)	1.40 (0.09)	0.98 (0.07)	0.99 (0.12)	1.09 (0.16)	1.34 (0.12)	1.63 (0.07)
	All	5.25 (6.53)	0.88 (0.06)	1.35 (0.27)	0.93 (0.08)	0.90 (0.20)	1.09 (0.25)	1.28 (0.23)	1.60 (0.10)
LOGO	Basic	6.38 (6.88)	0.84 (0.03)	1.53 (0.23)	1.05 (0.10)	1.15 (0.27)	1.37 (0.30)	1.55 (0.20)	1.58 (0.08)
	Acoustic	5.60 (6.99)	0.87 (0.02)	1.38 (0.33)	0.97 (0.09)	0.99 (0.21)	1.21 (0.27)	1.33 (0.25)	1.69 (0.04)
	Linguistic	5.70 (4.13)	0.84 (0.02)	1.42 (0.17)	0.99 (0.07)	1.07 (0.09)	1.17 (0.14)	1.42 (0.18)	1.67 (0.10)
	All	5.68 (7.85)	0.84 (0.03)	1.40 (0.39)	0.97 (0.11)	0.98 (0.22)	1.19 (0.27)	1.36 (0.26)	1.63 (0.05)
LOCO	Basic	6.70 (0.36)	0.88 (0.00)	1.75 (0.00)	1.23 (0.01)	1.19 (0.03)	1.53 (0.00)	1.59 (0.01)	1.84 (0.05)
	Acoustic	6.08 (3.24)	0.88 (0.01)	1.49 (0.16)	1.05 (0.07)	1.06 (0.05)	1.31 (0.16)	1.38 (0.12)	1.94 (0.00)
	Linguistic	6.35 (0.52)	0.89 (0.00)	1.55 (0.02)	1.06 (0.02)	1.12 (0.01)	1.29 (0.01)	1.48 (0.01)	1.86 (0.03)
	All	6.35 (2.85)	0.88 (0.00)	1.54 (0.22)	1.05 (0.08)	1.10 (0.05)	1.34 (0.14)	1.45 (0.11)	1.94 (0.00)

RHO: repeated hold-out, SKF: stratified k-fold, KF: k-fold, LOGO: leave-one group out, LOCO: leave-one context out.

**Table A3:** RMSE of random forest regression model.

Strategy	Features	CQ	ARG	CF	CO	ITO	KE	SMU	SPST
RHO	Basic	4.62 (0.10)	0.79 (0.00)	1.14 (0.00)	0.91 (0.00)	0.84 (0.01)	1.02 (0.01)	1.09 (0.01)	1.42 (0.01)
	Acoustic	4.61 (0.10)	0.77 (0.00)	1.15 (0.01)	0.93 (0.00)	0.81 (0.01)	1.00 (0.00)	1.14 (0.01)	1.46 (0.01)
	Speech	4.96 (0.10)	0.75 (0.00)	1.26 (0.01)	0.89 (0.00)	0.94 (0.00)	1.03 (0.00)	1.23 (0.01)	1.49 (0.01)
	All	4.50 (0.11)	0.74 (0.00)	1.12 (0.01)	0.88 (0.00)	0.81 (0.00)	0.96 (0.00)	1.10 (0.01)	1.45 (0.01)
SKF	Basic	4.68 (0.02)	0.76 (0.00)	1.13 (0.00)	0.90 (0.00)	0.84 (0.00)	1.01 (0.00)	1.11 (0.00)	1.41 (0.01)
	Acoustic	4.60 (0.22)	0.78 (0.00)	1.14 (0.00)	0.93 (0.00)	0.80 (0.00)	0.99 (0.01)	1.10 (0.00)	1.44 (0.00)
	Speech	4.82 (0.08)	0.74 (0.00)	1.26 (0.00)	0.90 (0.00)	0.92 (0.00)	1.01 (0.00)	1.20 (0.00)	1.47 (0.01)
	All	4.48 (0.04)	0.75 (0.00)	1.12 (0.01)	0.89 (0.00)	0.80 (0.00)	0.94 (0.00)	1.08 (0.00)	1.44 (0.00)
KF	Basic	5.11 (3.67)	0.80 (0.05)	1.24 (0.13)	0.95 (0.06)	0.91 (0.18)	1.09 (0.16)	1.19 (0.10)	1.43 (0.11)
	Acoustic	4.63 (3.16)	0.78 (0.05)	1.19 (0.10)	0.93 (0.07)	0.84 (0.08)	0.98 (0.23)	1.16 (0.16)	1.48 (0.11)
	Speech	4.87 (3.36)	0.75 (0.04)	1.24 (0.11)	0.90 (0.05)	0.90 (0.15)	1.01 (0.16)	1.23 (0.12)	1.47 (0.11)
	All	4.65 (3.31)	0.73 (0.04)	1.19 (0.10)	0.86 (0.05)	0.84 (0.09)	0.97 (0.16)	1.17 (0.17)	1.45 (0.13)
LOGO	Basic	6.21 (8.16)	0.77 (0.03)	1.42 (0.23)	1.02 (0.07)	1.11 (0.26)	1.38 (0.25)	1.41 (0.19)	1.55 (0.10)
	Acoustic	5.85 (8.06)	0.79 (0.03)	1.36 (0.29)	1.00 (0.09)	1.10 (0.20)	1.20 (0.39)	1.35 (0.31)	1.50 (0.12)
	Speech	5.28 (3.60)	0.74 (0.03)	1.33 (0.16)	0.92 (0.06)	1.01 (0.12)	1.09 (0.13)	1.31 (0.11)	1.57 (0.12)
	All	5.79 (6.88)	0.74 (0.04)	1.39 (0.26)	0.91 (0.08)	1.18 (0.22)	1.20 (0.32)	1.37 (0.29)	1.51 (0.11)
LOCO	Basic	6.01 (2.98)	0.83 (0.00)	1.57 (0.00)	1.07 (0.04)	1.07 (0.08)	1.40 (0.03)	1.51 (0.01)	1.78 (0.03)
	Acoustic	6.63 (1.39)	0.84 (0.00)	1.54 (0.06)	1.07 (0.04)	1.07 (0.02)	1.41 (0.07)	1.48 (0.05)	1.72 (0.07)
	Speech	5.78 (1.85)	0.78 (0.00)	1.48 (0.06)	0.99 (0.03)	1.09 (0.04)	1.24 (0.01)	1.39 (0.03)	1.83 (0.05)
	All	6.50 (0.81)	0.80 (0.00)	1.52 (0.04)	1.01 (0.04)	1.11 (0.04)	1.37 (0.04)	1.47 (0.05)	1.72 (0.06)

**Table A4:** RMSE of ada boost regression model.

Strategy	Features	CQ	ARG	CF	CO	ITO	KE	SMU	SPST
RHO	Basic	4.68 (0.12)	0.77 (0.00)	1.14 (0.01)	0.89 (0.00)	0.85 (0.01)	1.03 (0.01)	1.09 (0.01)	1.43 (0.01)
	Acoustic	4.70 (0.13)	0.77 (0.00)	1.19 (0.01)	0.94 (0.01)	0.84 (0.01)	1.03 (0.01)	1.17 (0.01)	1.50 (0.01)
	Speech	4.79 (0.10)	0.74 (0.00)	1.22 (0.01)	0.87 (0.00)	0.91 (0.00)	1.00 (0.00)	1.19 (0.01)	1.47 (0.01)
	All	4.52 (0.14)	0.74 (0.00)	1.16 (0.01)	0.89 (0.00)	0.85 (0.01)	0.99 (0.01)	1.14 (0.01)	1.53 (0.01)
SKF	Basic	4.73 (0.01)	0.75 (0.00)	1.12 (0.00)	0.88 (0.00)	0.86 (0.00)	1.02 (0.00)	1.11 (0.00)	1.44 (0.01)
	Acoustic	4.70 (0.32)	0.76 (0.00)	1.15 (0.01)	0.95 (0.00)	0.83 (0.00)	1.02 (0.01)	1.13 (0.00)	1.49 (0.00)
	Speech	4.79 (0.09)	0.74 (0.00)	1.22 (0.00)	0.88 (0.00)	0.89 (0.00)	0.99 (0.00)	1.17 (0.00)	1.46 (0.01)
	All	4.50 (0.07)	0.73 (0.00)	1.13 (0.01)	0.89 (0.00)	0.83 (0.00)	0.95 (0.00)	1.09 (0.01)	1.48 (0.00)
KF	Basic	5.00 (4.39)	0.77 (0.05)	1.24 (0.19)	0.90 (0.06)	0.88 (0.21)	1.09 (0.21)	1.20 (0.15)	1.42 (0.18)
	Acoustic	4.77 (3.71)	0.77 (0.04)	1.20 (0.14)	0.92 (0.10)	0.84 (0.13)	0.99 (0.24)	1.20 (0.22)	1.43 (0.25)
	Speech	4.67 (3.79)	0.73 (0.04)	1.21 (0.13)	0.87 (0.05)	0.86 (0.17)	0.98 (0.16)	1.17 (0.14)	1.45 (0.12)
	All	4.66 (3.69)	0.73 (0.04)	1.21 (0.18)	0.86 (0.07)	0.85 (0.14)	0.96 (0.18)	1.20 (0.24)	1.43 (0.26)
LOGO	Basic	6.02 (8.40)	0.76 (0.03)	1.42 (0.30)	0.98 (0.09)	1.05 (0.32)	1.35 (0.29)	1.41 (0.26)	1.48 (0.20)
	Acoustic	5.89 (8.64)	0.79 (0.03)	1.45 (0.40)	0.97 (0.13)	1.07 (0.28)	1.17 (0.44)	1.40 (0.40)	1.47 (0.21)
	Speech	4.98 (3.89)	0.72 (0.03)	1.29 (0.18)	0.90 (0.06)	0.95 (0.14)	1.05 (0.14)	1.24 (0.14)	1.53 (0.18)
	All	5.70 (8.37)	0.73 (0.03)	1.45 (0.37)	0.91 (0.13)	1.13 (0.26)	1.19 (0.42)	1.40 (0.38)	1.48 (0.24)
LOCO	Basic	6.11 (2.83)	0.82 (0.00)	1.69 (0.00)	1.05 (0.05)	1.09 (0.05)	1.46 (0.03)	1.55 (0.02)	1.86 (0.09)
	Acoustic	6.88 (1.55)	0.85 (0.00)	1.68 (0.02)	1.07 (0.06)	1.14 (0.01)	1.45 (0.12)	1.61 (0.04)	1.79 (0.12)
	Speech	5.47 (1.69)	0.75 (0.00)	1.47 (0.05)	0.96 (0.04)	1.05 (0.03)	1.23 (0.00)	1.33 (0.03)	1.81 (0.08)
	All	6.85 (1.03)	0.80 (0.00)	1.71 (0.00)	1.04 (0.07)	1.15 (0.02)	1.42 (0.13)	1.61 (0.03)	1.87 (0.07)

**Table A5:** RMSE of gradient boost regression model.

Strategy	Features	CQ	ARG	CF	CO	ITO	KE	SMU	SPST
RHO	Basic	4.87 (0.14)	0.84 (0.01)	1.36 (0.02)	1.04 (0.01)	0.88 (0.01)	1.09 (0.01)	1.25 (0.01)	1.63 (0.02)
	Acoustic	4.84 (0.13)	0.84 (0.00)	1.35 (0.01)	1.04 (0.01)	0.85 (0.01)	1.06 (0.01)	1.22 (0.02)	1.66 (0.02)
	Speech	4.92 (0.13)	0.78 (0.00)	1.29 (0.01)	0.92 (0.01)	0.94 (0.01)	1.09 (0.01)	1.26 (0.01)	1.69 (0.02)
	All	4.69 (0.14)	0.82 (0.01)	1.25 (0.03)	1.05 (0.01)	0.86 (0.01)	1.01 (0.01)	1.16 (0.01)	1.66 (0.02)
SKF	Basic	4.96 (0.10)	0.83 (0.01)	1.39 (0.00)	1.05 (0.00)	0.88 (0.00)	1.12 (0.00)	1.21 (0.00)	1.67 (0.00)
	Acoustic	4.75 (0.10)	0.85 (0.00)	1.35 (0.00)	1.05 (0.00)	0.85 (0.00)	1.09 (0.00)	1.21 (0.01)	1.68 (0.00)
	Speech	4.94 (0.02)	0.82 (0.00)	1.38 (0.00)	0.94 (0.00)	0.93 (0.00)	1.11 (0.00)	1.26 (0.00)	1.69 (0.00)
	All	4.66 (0.07)	0.83 (0.00)	1.33 (0.00)	1.05 (0.00)	0.85 (0.00)	1.01 (0.00)	1.18 (0.01)	1.67 (0.00)
KF	Basic	5.22 (5.86)	0.82 (0.07)	1.31 (0.32)	0.98 (0.14)	0.89 (0.22)	1.06 (0.20)	1.19 (0.22)	1.55 (0.39)
	Acoustic	4.77 (4.81)	0.86 (0.06)	1.23 (0.36)	0.98 (0.14)	0.83 (0.17)	1.05 (0.23)	1.22 (0.21)	1.55 (0.45)
	Speech	4.68 (4.36)	0.80 (0.04)	1.25 (0.27)	0.91 (0.09)	0.88 (0.21)	1.06 (0.22)	1.18 (0.23)	1.58 (0.39)
	All	4.60 (5.02)	0.79 (0.04)	1.29 (0.33)	0.98 (0.14)	0.82 (0.18)	1.01 (0.21)	1.21 (0.21)	1.55 (0.43)
LOGO	Basic	5.67 (7.47)	0.89 (0.04)	1.31 (0.38)	1.00 (0.16)	0.99 (0.27)	1.27 (0.60)	1.24 (0.24)	1.57 (0.34)
	Acoustic	5.73 (8.20)	0.89 (0.03)	1.30 (0.37)	1.00 (0.16)	1.02 (0.21)	1.27 (0.63)	1.32 (0.28)	1.60 (0.35)
	Speech	5.15 (5.05)	0.87 (0.05)	1.28 (0.33)	0.90 (0.11)	0.93 (0.23)	1.24 (0.63)	1.23 (0.23)	1.59 (0.37)
	All	5.45 (6.90)	0.81 (0.04)	1.48 (0.34)	1.00 (0.16)	1.07 (0.22)	1.24 (0.64)	1.27 (0.26)	1.58 (0.34)
LOCO	Basic	5.94 (4.00)	0.88 (0.00)	1.75 (0.02)	1.11 (0.09)	1.01 (0.11)	1.32 (0.21)	1.39 (0.09)	1.88 (0.21)
	Acoustic	6.15 (3.61)	0.85 (0.00)	1.66 (0.06)	1.11 (0.09)	1.07 (0.05)	1.36 (0.15)	1.41 (0.09)	1.83 (0.26)
	Speech	5.51 (3.07)	0.80 (0.00)	1.50 (0.13)	0.97 (0.07)	1.03 (0.06)	1.16 (0.12)	1.32 (0.12)	1.87 (0.20)
	All	5.83 (2.72)	0.84 (0.00)	1.67 (0.06)	1.11 (0.09)	1.06 (0.06)	1.26 (0.13)	1.37 (0.10)	1.82 (0.26)

**Table A6:** RMSE of neural network regression model.

Strategy	Features	CQ	ARG	CF	CO	ITO	KE	SMU	SPST
RHO	Basic	4.84 (0.11)	0.81 (0.01)	1.21 (0.01)	0.94 (0.01)	0.91 (0.01)	1.06 (0.01)	1.16 (0.01)	1.43 (0.01)
	Acoustic	5.72 (0.15)	0.92 (0.01)	1.45 (0.01)	1.17 (0.01)	1.16 (0.01)	1.32 (0.02)	1.48 (0.01)	1.66 (0.02)
	Speech	4.86 (0.09)	0.76 (0.00)	1.22 (0.00)	0.90 (0.01)	0.91 (0.00)	1.04 (0.00)	1.20 (0.01)	1.47 (0.01)
	All	5.34 (0.13)	0.92 (0.01)	1.46 (0.01)	1.08 (0.01)	1.11 (0.01)	1.33 (0.01)	1.37 (0.01)	1.69 (0.03)
SKF	Basic	5.16 (0.07)	0.78 (0.00)	1.20 (0.01)	0.92 (0.00)	0.91 (0.00)	1.07 (0.01)	1.17 (0.00)	1.41 (0.00)
	Acoustic	6.19 (0.17)	0.90 (0.01)	1.52 (0.04)	1.15 (0.00)	1.17 (0.01)	1.37 (0.02)	1.40 (0.04)	1.77 (0.01)
	Speech	4.89 (0.04)	0.74 (0.00)	1.20 (0.00)	0.88 (0.00)	0.91 (0.00)	1.02 (0.00)	1.19 (0.00)	1.50 (0.00)
	All	5.31 (0.03)	1.00 (0.00)	1.46 (0.01)	1.12 (0.00)	1.08 (0.00)	1.37 (0.00)	1.45 (0.04)	1.89 (0.04)
KF	Basic	5.27 (3.84)	0.80 (0.03)	1.35 (0.21)	0.98 (0.06)	0.91 (0.15)	1.11 (0.17)	1.30 (0.17)	1.49 (0.10)
	Acoustic	6.21 (4.76)	0.97 (0.04)	1.49 (0.13)	1.19 (0.14)	1.10 (0.12)	1.51 (0.12)	1.50 (0.21)	1.83 (0.07)
	Speech	4.71 (3.69)	0.74 (0.04)	1.21 (0.12)	0.89 (0.06)	0.88 (0.15)	1.02 (0.16)	1.18 (0.14)	1.56 (0.13)
	All	5.82 (3.08)	0.97 (0.02)	1.53 (0.12)	1.15 (0.05)	1.10 (0.10)	1.24 (0.07)	1.43 (0.10)	1.73 (0.08)
LOGO	Basic	6.29 (9.21)	0.82 (0.03)	1.39 (0.19)	1.05 (0.09)	1.15 (0.22)	1.29 (0.17)	1.44 (0.16)	1.56 (0.07)
	Acoustic	6.61 (3.99)	1.02 (0.00)	1.49 (0.23)	1.16 (0.04)	1.18 (0.10)	1.42 (0.12)	1.60 (0.13)	1.83 (0.03)
	Speech	4.95 (3.51)	0.76 (0.02)	1.32 (0.15)	0.91 (0.07)	0.96 (0.13)	1.10 (0.15)	1.31 (0.16)	1.49 (0.13)
	All	6.47 (2.41)	0.94 (0.04)	1.62 (0.19)	1.13 (0.13)	1.17 (0.16)	1.46 (0.14)	1.64 (0.14)	1.84 (0.05)
LOCO	Basic	5.91 (2.11)	1.02 (0.00)	1.98 (0.27)	1.06 (0.05)	1.26 (0.03)	1.37 (0.04)	1.57 (0.00)	1.64 (0.04)
	Acoustic	6.42 (2.77)	0.97 (0.00)	1.74 (0.00)	1.40 (0.01)	1.25 (0.27)	1.51 (0.02)	1.67 (0.01)	1.95 (0.06)
	Speech	5.50 (2.29)	0.78 (0.00)	1.46 (0.04)	1.05 (0.02)	1.07 (0.05)	1.19 (0.02)	1.37 (0.05)	1.72 (0.06)
	All	6.39 (0.65)	0.97 (0.02)	1.53 (0.00)	1.37 (0.01)	1.25 (0.14)	1.69 (0.15)	1.57 (0.04)	1.88 (0.00)
